# Absence of Modulation in Interhemispheric Inhibition During Sustained Muscle Contraction as Measured by Ipsilateral Silent Period

**DOI:** 10.7759/cureus.96481

**Published:** 2025-11-10

**Authors:** Satoshi Kudoh, Yuya Matsuda, Rin Kosuge, Mayu Akaiwa, Takeshi Sasaki, Hidekazu Saito, Kazuhiro Sugawara

**Affiliations:** 1 Department of Rehabilitation, Sapporo Hakuyokai Hospital, Sapporo, JPN; 2 Graduate School of Health Sciences, Sapporo Medical University, Sapporo, JPN; 3 Department of Human Health Sciences, Graduate School of Medicine, Kyoto University, Kyoto, JPN; 4 Department of Physical Therapy, School of Health Sciences, Sapporo Medical University, Sapporo, JPN; 5 Department of Occupational Therapy, School of Health Sciences, Sapporo Medical University, Sapporo, JPN

**Keywords:** electromyography, interhemispheric inhibition, ipsilateral silent period, motor evoked potentials, transcranial magnetic stimulation

## Abstract

Background: Interhemispheric inhibition (IHI) can be assessed using transcranial magnetic stimulation (TMS). The ipsilateral silent period (ISP), which represents IHI from the primary motor areas (M1) on the TMS stimulation side to the M1 on the opposite side of the TMS stimulation, is elicited. The variability in ISP associated with changes in contraction intensity at 30% and 50% of maximum voluntary contraction (MVC) and the muscle of the contralateral limb remains unclear.

Materials and methods: This study investigated the influence of these variables on ISPs. We performed electromyography monitoring for ISP and evaluated motor evoked potentials (MEPs) and contraction force during motor tasks in 15 right-handed healthy adults. Measurements involved the bilateral first dorsal interosseous and the right biceps brachii muscles. After identifying the optimal TMS target (hot spot) and establishing the resting motor threshold, 20 TMS pulses were applied in each condition.

Results: There was no interaction or main effect on ISP duration under any condition, while MEP displayed significant interaction and main effects for contraction intensity and muscle type. We found no variations in ISP across motor tasks in this study.

Conclusions: We observed no ISP alterations despite changes in M1 excitability, suggesting that other brain regions, such as the supplementary motor area (SMA), may modulate IHI.

## Introduction

The smooth motion of unilateral or bilateral limbs in humans requires the coordination of bilateral hemispheric activities, with the corpus callosum playing an important role in such coordination [1]. Interhemispheric inhibition (IHI) via the corpus callosum is considered necessary for each hemisphere to inhibit each other and prevent interference between them [2]. Interestingly, the imbalance of IHI from the non-injured to the injured hemisphere can inhibit the movement of the affected limb, leading to a poor prognosis [3,4]. In addition, among patients with severe motor paralysis, those with subsequently improved motor function on the affected side showed a decrease in IHI from the non-injured to the injured hemisphere [5]. IHI can be measured using transcranial magnetic stimulation (TMS) in both healthy people and stroke patients [6,7].

TMS is a method to examine neuroplasticity, corticospinal excitability, and corpus callosum conduction by noninvasively stimulating the brain [8]. The evaluation of IHI via TMS consists of two paired-pulse TMS to stimulate bilateral primary motor areas (M1) and single-pulse TMS to stimulate the unilateral M1 that measures electromyography (EMG) changes to the ipsilateral limb. Paired-pulse TMS is difficult to implement in clinical practice because it requires two TMS devices. Nevertheless, another method allows measuring the ipsilateral silent period (ISP) as an evaluation index of IHI during the sustained muscle contraction of one limb using single-pulse TMS [6,9]. ISP measures a temporary EMG interruption or disappearance in the "target muscle" by stimulating the ipsilateral M1 with single-pulse TMS. At this time, the "target muscle" is continuously contracting. ISPs are typically quantified by duration that provide a measure of the suppression of the EMG. Greater duration is interpreted as greater IHI [7]. Recent TMS-based studies have refined our understanding of how IHI dynamically adapts during motor tasks [10].

When implementing evaluation and exercise therapy in rehabilitation, it is important to evaluate IHI during muscle contraction. In previous studies, ISP increased when the target muscle and contralateral homologous muscle were simultaneously contracted, compared to the contraction of the target muscle alone [11]. Therefore, it is clinically important to understand how muscle contraction and its strength change IHI in stroke patients for evaluation, prognosis prediction, and treatment decisions. However, there are few reports on how ISP is modulated by contraction intensity and muscle type (proximal vs. distal) in the contralateral limb, and the underlying mechanisms remain poorly understood. Previous studies have reported inconsistent findings and were conducted with relatively small sample sizes [11]. Clarifying this relationship is important for understanding the neural mechanisms of IHI and for improving the interpretation of ISP as a clinical and physiological index. Therefore, the present study aimed to elucidate the modulation of ISP by systematically varying contraction intensity and muscle type during sustained muscle contraction.

This study aimed to investigate how muscle contraction in the contralateral limb affects the ISP during the sustained contraction of the target muscle. In particular, we focused on the changes in ISP by changing the task conditions of contracting strength and muscles. This study was designed to elucidate a poorly understood aspect of motor neurophysiology, the precise modulation of the ISP by different contraction intensities and muscle types. We hypothesized that the ipsilateral M1 excitability and ISP increased when the contraction strength of the muscle in the limb contralateral to the target muscle increased; similarly, the ipsilateral M1 excitability and ISP increased more with the homologous muscle of the target muscle contraction than with the non-synonymous muscle contraction.

## Materials and methods

Subjects

We determined the minimum sample size using the G*power software (Ver. 3.1.9.7, Heinrich-Heine-Universität Düsseldorf, Düsseldorf, Germany) from partial η-squared. An a priori power analysis was conducted using G*Power 3.1 based on a large effect size from prior work (η² = 0.85) [7,12], with α = 0.05 and power (1 − β) = 0.80, indicating a required sample size of n = 15 for the planned repeated-measures analysis of variance (ANOVA). Thus, 15 right-handed healthy adults (mean age 25.3 years; SD 2.0 years; 10 men and five women) were included in the study. Handedness was assessed using the Edinburgh Handedness Inventory, and all participants scored above the threshold indicating right-handed dominance. Individuals with a history of neurological insult (e.g., head injury, history of migraines, epilepsy) or taking medication with contraindications for TMS were excluded [13,14]. All participants were free from musculoskeletal or psychiatric disorders and reported no regular participation in competitive sports or structured strength training programs. None had prior experience with TMS experiments. All participants provided written informed consent. The study was conducted at Sapporo Medical University, Sapporo, Japan, and conformed to the Declaration of Helsinki and the Code of Ethics of the World Medical Association. Approval was obtained from the Ethics Committee of Sapporo Medical University (approval number: 2-1-91).

Experimental procedure

**Figure 1:**
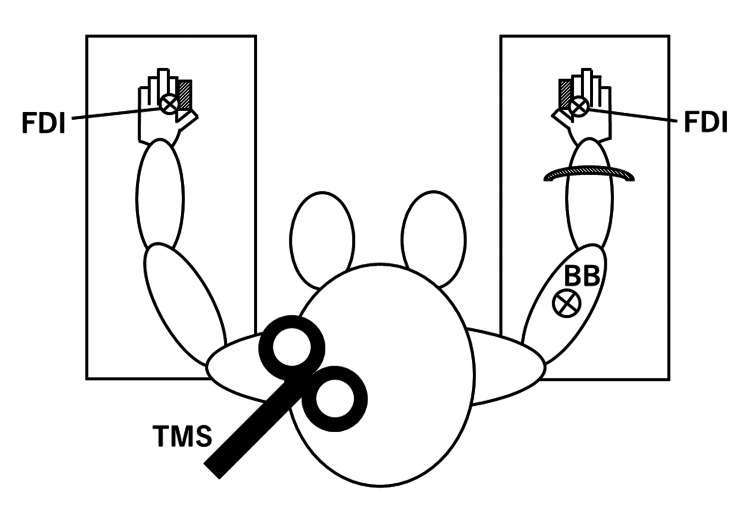
Experimental design Bilateral shoulder joints were abducted at 45°, bilateral elbow joints flexed at 90°, and bilateral forearms pronated at 45°. FDI: first dorsal interosseous; BB: biceps brachii; TMS: transcranial magnetic stimulation This figure was created by the authors using Microsoft PowerPoint (Microsoft Corporation, Redmond, WA, USA). No external copyrighted materials were used.

During the experiments, subjects were seated in a comfortable chair with their arms fully supported (Figure 1). At this time, the bilateral shoulder joints were abducted at 45°, bilateral elbow joints flexed at 90°, and bilateral forearms pronated at 45°. In this experiment, TMS stimulation intensity was determined based on the resting motor threshold (RMT). Afterwards, we measured ISP duration during each motor task.

EMG recording

Surface EMG activity was recorded simultaneously from the bilateral first dorsal interosseous (FDI) muscles and right biceps brachii (BB), whereas the right FDI was adopted as a distal and homologous muscle and the right BB was adopted as a proximal and non-homologous muscle. Disposable electrodes were placed in a tendon-belly arrangement for FDI and the middle of the upper arm for BB. After amplifying the signal, EMG signals were sampled at 2 kHz and band-pass filtered at 10-500 Hz.

TMS procedure

TMS of the left motor cortex was performed using a coil and Magstim 200 stimulator (The Magstim Company Limited, Dyfed, UK). The coil was placed at the optimal position (hot spot) of the right FDI for eliciting motor evoked potential (MEP). The handle of the coil pointed backwards and was 45° to the midsagittal line [9,11,15,16]. RMT was defined as the minimum stimulus intensity, which elicited MEPs >50 μV in ≥5 out of 10 consecutive trials [7] in the right FDI. In all conditions, ISP was elicited by stimulation at 130% RMT. TMS stimulation was randomly administered at intervals of 10-15 seconds. The examiner checked on the monitor whether the contraction strength was maintained during each task, and in case of >5% deviation from the specified EMG, TMS stimulation was not performed.

Experimental task

Before measuring ISP, we measured EMG (mV) during maximum voluntary contraction (MVC) of both index finger abduction and right elbow flexion (100 %EMG). All subjects performed the following tasks: Condition 1, rest (right upper limb contraction at 0 %EMG); Condition 2, right index finger abduction at 30 %EMG; Condition 3, right index finger abduction at 50 %EMG; Condition 4, right elbow flexion at 30 %EMG; and Condition 5, right elbow flexion at 50 %EMG. The muscle contraction strength for each task was determined by referring to the EMG normalized by 100 %EMG. In this study, EMG values (100 %EMG) were normalized to each participant's MVC. The left hand performed isometric contractions of index finger abduction at 20 %EMG of MVC in under all task conditions. For the right hand, all five conditions listed below were performed 20 times. The contraction intensity levels were set at 30% and 50% of MVC for both the FDI and BB muscles. These intensity levels were chosen to represent moderate and relatively high submaximal effort, allowing stable contraction without inducing fatigue. The order of task conditions was manually randomized by the examiner for each participant to minimize order effects. At this time, the subjects adjusted the contraction strength based on the guidelines displayed on the monitor. The examiner checked the monitor to see if the subject was performing any muscle contractions other than those required for the task. If any muscle contractions other than those required for the task were occurring, the examiner instructed the subjects not to perform the contractions.

Data analysis

In this study, electromyographic data were analyzed using LabChart (Version 8.1.21, ADInstruments, Dunedin, New Zealand). After rectifying and averaging the 20 EMGs measured in each task, the following analysis was performed for each measured variable.

ISP

**Figure 2:**
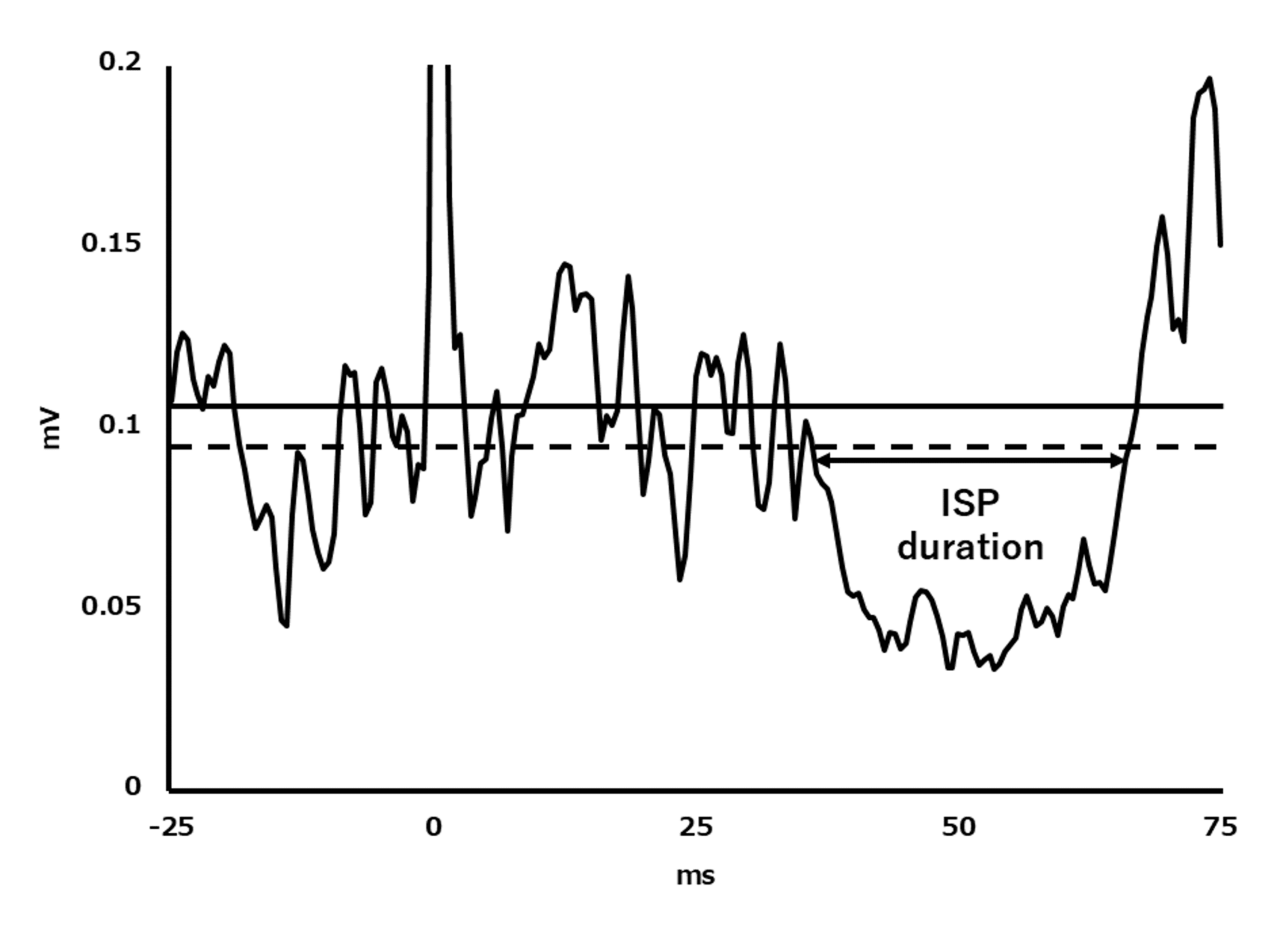
Example trace showing the average of 20 traces with the resulting silent period The horizontal solid line indicates mean pre-stimulus EMG and the dotted line the lower variation limits of the EMG signal. ISP: ipsilateral silent period; EMG: electromyography

The ISP was calculated by using an objective, graphical method described in detail by Garvey and colleagues [17]. Upper and lower variation limits of the EMG signal were calculated by determining the mean consecutive difference (MCD) of EMG data points 100 ms before stimulation: mean pre-stimulus EMG ± (|MCD| × 1.77). ISP onset and offset were identified using the following criteria: (1) time of onset, the first of five consecutive data points below the lower variation limit; (2) all subsequent data points were considered part of the ISP until a return of sustained EMG activity; and (3) time of offset, the first data point above the lower variation limit if ≥50% data points in the following 5 ms window were also above the variation limit. ISP duration (ms) was calculated as ISP offset-ISP onset [18] (Figure 2).

%EMG

The MVC of bilateral index finger abduction and right elbow flexion was performed for seven seconds, and the analysis interval was three seconds, excluding two seconds before and after. The muscle activity at that time was considered 100 %EMG. EMG signals were first rectified, before calculating the integral EMG. All muscle contraction strengths set in the task were normalized to 100 %EMG and displayed on the monitor as a guideline. The average EMG of all trials was calculated for each condition. The analysis interval comprised from 100 ms before TMS stimulation to TMS stimulation. The %EMG for each condition was calculated using the following formula: $\%\text{EMG}=\text{average EMG of all trials}/\text{MVC}\times100\text{(\%)}$.

MEP

Peak-to-peak MEP amplitudes were calculated in the EMG derived from the right FDI after TMS stimulation.

Ipsilateral MEP (iMEP)

To investigate the presence of ipsilateral MEP (iMEP), iMEP was calculated from EMG derived from the left FDI. An iMEP was deemed to be present if the post-stimulus EMG exceeded the pre-stimulus mean by >1 SD for ≥5 ms [9].

Statistical analysis

Statistical analyses were performed using IBM SPSS Statistics for Windows, V. 25.0 (IBM Corp., Armonk, NY, USA). To minimize bias, data analysis was performed by an investigator who was blinded to the task condition labels. The examiner who performed TMS and EMG recordings was not blinded to the conditions. The normality of data was attested by the Shapiro-Wilk test. A two-way repeated-measures ANOVA (contraction muscle factor (FDI and BB) × contraction strength factor (0%, 30%, 50%)) was used to compare mean ISP duration and MEP amplitude. Post hoc analyses were performed with Bonferroni's methods. A t-test was used to assess differences in %EMG of the right FDI and right BB between contraction strengths (30% and 50%). Moreover, differences across Conditions 1-5 for %EMG of the left FDI were assessed using one-way repeated-measures ANOVA. To test the sphericity condition, Mauchly's test was used. Additionally, the p-values were adjusted for possible deviations using the Greenhouse-Geisser corrections. The statistical significance was set at p<0.05.

## Results

％EMG

The %EMG of the right FDI 50 %EMG condition was significantly larger than the 30 %EMG condition of the right FDI (t = 5.69; df = 28; p < 0.01). The %EMG of the right BB 50 %EMG condition was significantly larger than the 30 %EMG condition of the right BB (t = 10.35; df = 28; p < 0.01; Table 1). The %EMG of the left FDI was approximately 19-21% across all conditions and showed no significant differences (F(2.534, 35.482) = 2.09; p = 0.13), confirming the stable contraction of the target muscle during each task condition (Table 1).

**Table 1:** MVC in each muscle and %EMG (mean ± SD) under different experimental conditions Statistical comparisons were performed using a t-test. *50 %EMG significantly greater than 30 %EMG within the same muscle (p < 0.01). MVC: maximum voluntary contraction; EMG: electromyography; FDI: first dorsal interosseous; BB: biceps brachii

Recorded muscle	MVC (mV)	50 %EMG (%)	30 %EMG (%)	20 %EMG (%)
Left FDI	0.59 ± 0.28	20.1 ± 2.3	19.6 ± 1.8	19.43 ± 1.2
Right FDI	0.52 ± 0.22	44.6 ± 9.1*	28.3 ± 5.6	-
Right BB	0.44 ± 0.22	48.2 ± 5.9*	29.0 ± 3.7	-

ISP duration

**Figure 3:**
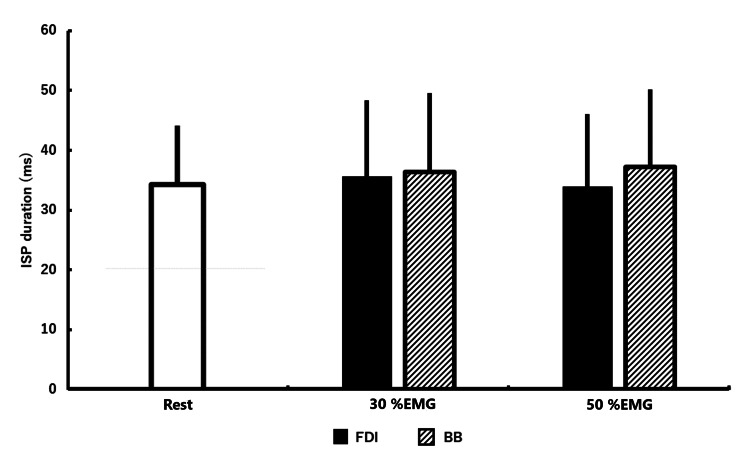
Average and standard deviation of ISP duration under all conditions Statistical comparisons were performed using two-way repeated-measures analysis of variance. ISP: ipsilateral silent period; EMG: electromyography; FDI: first dorsal interosseous; BB: biceps brachii

The ISP duration from each condition is shown in Table 2 and Figure 3.

**Table 2:** ISP duration and MEP amplitudes (mean ± SD) under different experimental conditions Statistical comparisons were performed using two-way repeated-measures analysis of variance followed by the Bonferroni post hoc tests. *MEP amplitude significantly greater than that at rest (p < 0.01) in post hoc tests. No significant differences were found for ISP duration. ISP: ipsilateral silent period; MEP: motor evoked potential; EMG: electromyography

Experimental conditions	ISP duration (ms)	MEP amplitudes (mV)
Rest	35.67 ± 10.67	1.94 ± 0.88
Right index finger abduction at 30 %EMG	35.67 ± 12.46	5.68 ± 2.67*
Right index finger abduction at 50 %EMG	33.87 ± 11.80	6.15 ± 1.60*
Right elbow flexion at 30 %EMG	36.33 ± 12.90	2.36 ± 2.47*
Right elbow flexion at 50 %EMG	37.20 ± 12.63	3.15 ± 2.12*

Two-way repeated-measures ANOVA revealed no significant contraction muscle × contraction strength interaction and no main effect of any factor (contraction muscle: F(1, 14) = 0.05, p = 0.83, and partial η^2^ = 0.03; contraction strength: F(2, 28) = 0.48, p = 0.64, and partial η^2^ = 0.33) (Figure 3).

MEP

**Figure 4:**
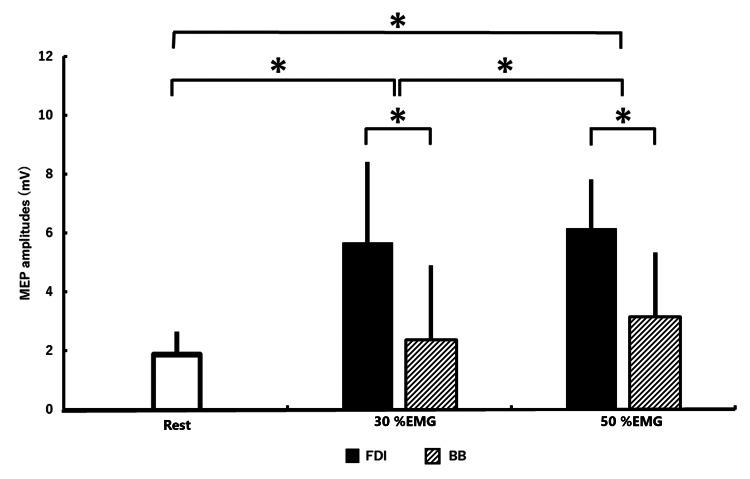
Average and standard deviation of MEP amplitude under all conditions Statistical comparisons were performed using two-way repeated-measures analysis of variance followed by the Bonferroni post hoc tests. *MEP amplitudes under the 30% and 50% contraction conditions significantly greater than those at rest (p < 0.01). MEP: motor evoked potential; EMG: electromyography; FDI: first dorsal interosseous; BB: biceps brachii

The MEP amplitudes from each condition are shown in Table 2 and Figure 4.

Two-way repeated-measures ANOVA revealed a significant contraction muscle × contraction strength interaction (F(2, 28) = 14.56; p < 0.01) and a main effect of the contraction muscle factor (F(1, 14) = 14.56; p < 0.01) and contraction strength factor (F(1, 17) = 41.82; p < 0.01). Post hoc analyses showed that the MEP amplitudes at the right FDI contraction condition were significantly greater than the right BB contraction condition (p < 0.01). In addition, MEP amplitudes at 30% (p < 0.01) and 50% (p < 0.01) contraction conditions were significantly greater than under rest conditions (Figure 4). Post hoc analyses showed that MEP amplitudes at 30% and 50% contraction conditions were significantly greater than those at rest (p < 0.01), as indicated by asterisks in Table 2 and Figure 4.

iMEP

iMEP appeared in four of the 15 subjects. Three of those did not exhibit iMEP in the right upper rest condition, but only in part of the right upper contraction condition. In addition, the ISP duration of the three subjects tended to be longer than the average ISP duration. No subject showed iMEP in all conditions.

## Discussion

This study evaluated IHI during the sustained contraction of a target muscle using TMS. The ISP was used as the index for assessing IHI. The study investigated how ISP changes in response to variations in task conditions, specifically the contraction strength and the identity of the muscle contracting in the contralateral limb, during the simultaneous sustained contraction of the target muscle.

We obtained the following main results: (1) ISP duration was not affected by differences in contraction muscle and contraction strength in the contralateral upper limb to the target muscle, and (2) MEP amplitude during right FDI contraction was significantly larger than during right BB contraction and significantly higher with increasing contraction strength.

Previous research has shown that ISP represents IHI via the corpus callosum [18]. Further, ISP during the isometric contraction of the upper limb contralateral to the target muscle reportedly increases compared with at rest [11]. In the present study, we focused on the difference in the contraction muscle in the contralateral upper limb and contraction strength as factors that increase M1 excitability. Animal [19] and magnetic resonance imaging studies in humans [20] have reported that corpus callosum fibers connect the bilateral M1 synapses, so we hypothesized that ISP would increase under contraction conditions of the right FDI, with the homologous muscle as the target muscle, than during right BB contraction. However, no changes in the ISP due to contraction muscles were observed in this study. Nor were changes in ISP during right upper limb contraction conditions observed compared to the resting condition on the right side, and no effect was observed due to differences in contraction strength. Since MEP reflects corticospinal excitability and interhemispheric motor regions, including those controlling the FDI, are connected via topographically organized callosal fibers [21], the MEP derived from the right FDI may indirectly reflect excitability within the left M1. As hypothesized, MEP significantly increased with the increasing contraction strength of the right upper limb, being significantly larger during right FDI contraction than during right BB contraction. Despite changes in M1 excitability in the left FDI region due to differences in contraction muscle and contraction strength, no change in ISP was observed from the left to right M1, suggesting that other factors, such as the activation of additional cortical areas, may have counteracted a potential decrease in ISP, resulting in the overall absence of significant ISP modulation observed in this study. This interpretation aligns with emerging evidence that ipsilateral motor cortical activity contributes actively to bimanual control rather than functioning solely as an inhibitory system [22].

Previous research has reported that long-latency IHI (LIHI) decreases during different motor tasks [23]. LIHI and short-latency IHI (SIHI) are IHI indices that can be derived by paired-pulse TMS by comparing the amplitude of MEP derived from the contralateral limb when TMS stimulation is applied to M1 on one side and the amplitude of MEP derived when TMS stimulation is applied to M1 on both sides [24]. IHI with an interstimulus interval of less than 10 ms is defined as SIHI, whereas IHI with an interstimulus interval of 40-50 ms is defined as LIHI [9]. Morishita et al. showed that LIHI decreased in a task in which participants manipulated an object with chopsticks on one side of the limb compared to a task in which participants performed repetitive hand movements without using an object on one side [24]. Several previous studies have shown that SIHI and LIHI involve distinct neural pathways, whereas the inhibitory mechanisms underlying LIHI and the ISP appear to share similar transcallosal circuits [9,25]. Moreover, evidence from patients with callosal infarction indicates that LIHI depends on additional cortical or subcortical elements beyond the primary motor cortex and callosal fibers [26]. Talelli et al. [27] further demonstrated that LIHI decreases with increased activity in the supplementary motor area (SMA), while Hummel et al. [28] reported that SMA activation intensifies during complex finger-sequence tasks, suggesting a functional link between SMA engagement and the modulation of IHI. Sadato et al. focused on SMA during bilateral hand movements in complex tasks and reported that SMA activity is equivalent to that during unilateral movements when both sides perform similar movements, but increases when each limb performs different movements compared to when both sides perform similar movements or during unilateral movements [29]. The SMA has been reported to modulate IHI, and increased SMA activity during complex or strength-regulated tasks can lead to a reduction in LIHI [24,27]. Because the inhibitory mechanisms underlying LIHI and the ISP are thought to be similar [9,26], enhanced SMA activity would theoretically shorten ISP duration. However, in the present study, ISP did not change across conditions. One possible explanation is that the degree of SMA activation induced by our task (isometric contractions at 30-50% MVC) was insufficient to elicit measurable ISP modulation or that other cortical or subcortical networks, such as premotor or sensory inputs, compensated for SMA-related effects. In the bilateral motor task used in this study, sustained contraction strength differed between upper limbs, and it was necessary to constantly adjust the contraction strength during the task. Therefore, it is possible that the absence of differences in ISP between conditions was due to insufficient SMA activation or compensatory modulation by other cortical networks, resulting in no detectable change in IHI.

When comparing the results of the present study with those of Giovannelli et al., the previous study reported a significantly greater ISP when there was contralateral upper limb contraction compared to when there was none [11]. In contrast, the present study did not find a significant difference in ISP depending on the presence or absence of contralateral upper limb contraction during target muscle activation. Moreover, while previous studies demonstrated a tendency for ISP to increase as the contraction strength of the upper limb contralateral to the target muscle increased, the present study did not observe any such trend. This discrepancy raises the question of whether differences in experimental methodology, aside from the presence or absence of motor modulation as previously discussed, contributed to the divergent results, an issue that will be discussed in the following section.

The first methodological difference lies in the contraction intensity of the target muscle. While Giovannelli et al. instructed participants to contract at their maximum sustainable strength [11], the present study employed a contraction at 20 %EMG. In our preliminary experiments, we attempted to replicate the conditions used by Giovannelli et al.; however, in all participants, muscle activity was observed in muscles other than the target muscle under those conditions. One of the objectives of the present study was to investigate changes in ISP depending on the specific muscle being contracted. To achieve this, it was necessary to isolate the contraction to a single muscle, and thus, experimental conditions that induced the activation of other muscles had to be avoided. Furthermore, low-intensity contractions are recommended for ISP measurement to minimize fatigue [7]. Given that the experiment involved five different conditions, maintaining a consistent contraction intensity across all trials was more feasible under low-intensity exercise. Based on these considerations, the present study adopted a 20 %EMG contraction level, which differed from the approach used by Giovannelli et al. [11]. Previous research has reported that variations in the contraction intensity of the target muscle do not affect the duration of the ISP [16]. Therefore, it is unlikely that the reduced contraction intensity of the target muscle in this study influenced the ISP measurements. The second methodological difference is that while Giovannelli et al. used ISP area as the index [11], the present study employed ISP duration. The mechanisms underlying these different ISP metrics remain unclear. In the present study, due to the lower contraction intensity, the background EMG prior to TMS stimulation was lower than that reported by Giovannelli et al. [11]. Since using ISP area as an index can be subject to a ceiling effect in EMG suppression under such conditions, ISP duration, which is less influenced by background EMG, was selected instead [7]. Importantly, although the use of ISP duration was expected to increase the likelihood of detecting significant differences between conditions, no such differences were found. Taken together, the methodological differences, such as the contraction intensity of the target muscle and the method used to calculate ISP, are unlikely to account for the discrepancy between the present findings and those of previous studies. Instead, the presence or absence of motor modulation appears to be a more critical factor underlying the current results, suggesting that elements beyond the primary motor area M1 may have been involved.

In this study, iMEPs were observed in four out of 15 participants. iMEPs are elicited via the ipsilateral motor pathway under TMS [7]. The presence of iMEPs is associated with IHI and may influence the duration of the ISP. In our study, although iMEPs were present in some of the four subjects, their ISP durations tended to be longer than average and did not appear to shorten the ISP.

The experimental task did not control sensory input, which may have affected the results. Sensory input can induce excitatory changes in the M1 [29]. Somatosensory input from cutaneous or proprioceptive receptors has been shown to increase cortical excitability and modulate IHI by influencing transcallosal and intracortical inhibitory pathways. Therefore, variations in tactile or proprioceptive feedback between task conditions could have altered the excitatory-inhibitory balance within M1 and contributed to interindividual variability in ISP duration. In the motor task used in this study, the level of superficial sensory input may have varied depending on the specific conditions, potentially influencing M1 excitability. To better manage sensory input in future experiments, it would be important to stabilize the hand and wrist position to minimize cutaneous feedback and to use soft padding or fixation devices to ensure equivalent tactile stimulation across conditions. Alternatively, monitoring somatosensory evoked potential or applying mild local anesthesia could help quantify or reduce peripheral sensory influence, thereby isolating cortical contributions to ISP modulation. Moreover, EMG recordings were limited to the bilateral FDI muscles and the right BB. It is possible that contractions occurred in other muscles that were not monitored, which could also have contributed to increased M1 excitability. Such unmeasured muscle activity may have affected ISP duration in addition to the activation of the recorded muscles.

The present study has several limitations that should be acknowledged. First, the sample size was relatively small (n = 15), which may reduce statistical power and limit generalizability. Second, only distal upper limb muscles were examined, and the results may not extend to proximal or lower limb muscles. Third, participants were young, healthy adults with limited demographic diversity, which restricts clinical extrapolation to populations such as stroke patients. Fourth, the contraction intensities tested (30-50% MVC) represented a moderate range and might have been insufficient to elicit measurable ISP changes. Future studies should address these limitations by including larger and more diverse samples, examining different muscle groups, and testing a wider range of contraction strengths to clarify the neural mechanisms underlying ISP modulation. In addition, the present study included only young, right-handed healthy adults. This restricts the generalizability of our findings to aging or clinical populations, such as individuals with stroke, in whom interhemispheric imbalance is most clinically relevant. Furthermore, only ISP duration was analyzed in this study; future research could combine ISP duration and area measures to improve sensitivity to subtle interhemispheric inhibitory changes. Finally, the absence of concurrent neuroimaging (e.g., diffusion tensor imaging of the corpus callosum) or electroencephalography limited our ability to directly relate physiological measures of ISP to structural or network-level mechanisms. Future research integrating multimodal approaches, such as TMS, electroencephalogram (EEG), and neuroimaging, may further clarify the dynamic cortical networks underlying ISP modulation [22,30].

## Conclusions

This study aimed to investigate how muscle contraction in the contralateral limb affects the ISP, focusing on the effects of contraction strength and muscle type (proximal vs. distal) during the sustained contraction of the target muscle. Contrary to our hypothesis, that ISP duration would increase with greater contraction strength and with homologous muscle activation, we observed no significant modulation of ISP under any condition. These findings suggest that ISP may remain stable despite increases in M1 excitability, implying that other cortical areas, such as the SMA, may influence the modulation of IHI. Understanding the stability and cortical modulation of ISP provides important insight into interhemispheric balance, which has direct implications for motor recovery strategies in stroke rehabilitation. However, these conclusions should be interpreted within the limitations of the present design, including the small sample size and constrained task conditions, and future studies are needed to confirm and extend these findings in larger and more diverse populations.

## References

[1] Carson RG (2005). Neural pathways mediating bilateral interactions between the upper limbs. Brain Res Brain Res Rev.

[2] Fling BW, Peltier SJ, Bo J, Welsh RC, Seidler RD (2011). Age differences in interhemispheric interactions: callosal structure, physiological function, and behavior. Front Neurosci.

[3] Duque J, Hummel F, Celnik P, Murase N, Mazzocchio R, Cohen LG (2005). Transcallosal inhibition in chronic subcortical stroke. Neuroimage.

[4] Murase N, Duque J, Mazzocchio R, Cohen LG (2004). Influence of interhemispheric interactions on motor function in chronic stroke. Ann Neurol.

[5] Lin YL, Potter-Baker KA, Cunningham DA (2020). Stratifying chronic stroke patients based on the influence of contralesional motor cortices: an inter-hemispheric inhibition study. Clin Neurophysiol.

[6] Meyer BU, Röricht S, Gräfin von Einsiedel H, Kruggel F, Weindl A (1995). Inhibitory and excitatory interhemispheric transfers between motor cortical areas in normal humans and patients with abnormalities of the corpus callosum. Brain.

[7] Hupfeld KE, Swanson CW, Fling BW, Seidler RD (2020). TMS-induced silent periods: a review of methods and call for consistency. J Neurosci Methods.

[8] Barker AT, Jalinous R, Freeston IL (1985). Non-invasive magnetic stimulation of human motor cortex. Lancet.

[9] Chen R, Yung D, Li JY (2003). Organization of ipsilateral excitatory and inhibitory pathways in the human motor cortex. J Neurophysiol.

[10] Tian D, Izumi SI, Suzuki E (2021). Modulation of interhemispheric inhibition between primary motor cortices induced by manual motor imitation: a transcranial magnetic stimulation study. Brain Sci.

[11] Giovannelli F, Borgheresi A, Balestrieri F, Zaccara G, Viggiano MP, Cincotta M, Ziemann U (2009). Modulation of interhemispheric inhibition by volitional motor activity: an ipsilateral silent period study. J Physiol.

[12] Davidson T, Tremblay F (2013). Age and hemispheric differences in transcallosal inhibition between motor cortices: an ispsilateral silent period study. BMC Neurosci.

[13] Rossi S, Hallett M, Rossini PM, Pascual-Leone A (2009). Safety, ethical considerations, and application guidelines for the use of transcranial magnetic stimulation in clinical practice and research. Clin Neurophysiol.

[14] Wassermann EM (1998). Risk and safety of repetitive transcranial magnetic stimulation: report and suggested guidelines from the International Workshop on the Safety of Repetitive Transcranial Magnetic Stimulation, June 5-7, 1996. Electroencephalogr Clin Neurophysiol.

[15] Fleming MK, Newham DJ (2016). Reliability of transcallosal inhibition in healthy adults. Front Hum Neurosci.

[16] Kuo YL, Dubuc T, Boufadel DF, Fisher BE (2017). Measuring ipsilateral silent period: effects of muscle contraction levels and quantification methods. Brain Res.

[17] Garvey MA, Ziemann U, Becker DA, Barker CA, Bartko JJ (2001). New graphical method to measure silent periods evoked by transcranial magnetic stimulation. Clin Neurophysiol.

[18] Fling BW, Seidler RD (2012). Task-dependent effects of interhemispheric inhibition on motor control. Behav Brain Res.

[19] Rouiller EM, Babalian A, Kazennikov O, Moret V, Yu XH, Wiesendanger M (1994). Transcallosal connections of the distal forelimb representations of the primary and supplementary motor cortical areas in macaque monkeys. Exp Brain Res.

[20] Brocke J, Schmidt S, Irlbacher K, Cichy RM, Brandt SA (2008). Transcranial cortex stimulation and fMRI: electrophysiological correlates of dual-pulse BOLD signal modulation. Neuroimage.

[21] Wahl M, Lauterbach-Soon B, Hattingen E (2007). Human motor corpus callosum: topography, somatotopy, and link between microstructure and function. J Neurosci.

[22] Bundy DT, Leuthardt EC (2019). The cortical physiology of ipsilateral limb movements. Trends Neurosci.

[23] Groppa S, Oliviero A, Eisen A (2012). A practical guide to diagnostic transcranial magnetic stimulation: report of an IFCN committee. Clin Neurophysiol.

[24] Morishita T, Kubota S, Hirano M, Funase K (2014). Different modulation of short- and long-latency interhemispheric inhibition from active to resting primary motor cortex during a fine-motor manipulation task. Physiol Rep.

[25] Ferbert A, Priori A, Rothwell JC, Day BL, Colebatch JG, Marsden CD (1992). Interhemispheric inhibition of the human motor cortex. J Physiol.

[26] Li JY, Lai PH, Chen R (2013). Transcallosal inhibition in patients with callosal infarction. J Neurophysiol.

[27] Talelli P, Ewas A, Waddingham W, Rothwell JC, Ward NS (2008). Neural correlates of age-related changes in cortical neurophysiology. Neuroimage.

[28] Hummel F, Kirsammer R, Gerloff C (2003). Ipsilateral cortical activation during finger sequences of increasing complexity: representation of movement difficulty or memory load?. Clin Neurophysiol.

[29] Sadato N, Yonekura Y, Waki A, Yamada H, Ishii Y (1997). Role of the supplementary motor area and the right premotor cortex in the coordination of bimanual finger movements. J Neurosci.

[30] Momi D, Neri F, Coiro G (2020). Cognitive enhancement via network-targeted cortico-cortical associative brain stimulation. Cereb Cortex.

